# mTOR Modulates Intercellular Signals for Enlargement and Infiltration in Glioblastoma Multiforme

**DOI:** 10.3390/cancers12092486

**Published:** 2020-09-02

**Authors:** Larisa Ryskalin, Francesca Biagioni, Paola Lenzi, Alessandro Frati, Francesco Fornai

**Affiliations:** 1 Department of Translational Research and New Technologies in Medicine and Surgery, University of Pisa, Via Roma 55, 56126 Pisa, Italy; larisa.ryskalin@unipi.it (L.R.); paola.lenzi@unipi.it (P.L.); 2 Istituto di Ricovero e Cura a Carattere Scientifico Neuromed, Via Atinense 18, 86077 Pozzilli (IS), Italy; francesca.biagioni@neuromed.it (F.B.); alessandro.frati@uniroma1.it (A.F.)

**Keywords:** glioma cancer stem cells, extracellular vesicles, exosomes, cell-to-cell communication, tumor microenvironment, GSC niche

## Abstract

**Simple Summary:**

Glioblastoma multiforme (GBM) is the most aggressive and lethal primary brain tumor. Emerging evidence indicate the multi-faceted role of extracellular vesicles (EVs) in GBM growth and proliferation. In fact, GBM-derived EVs can alter the phenotype of GBM-associated parenchymal cells; thus, promoting tumor growth, angiogenesis, and immune evasion. Remarkably, among several pathways that are frequently deregulated in GBM, mammalian Target of Rapamycin (mTOR) up-regulation, and subsequent autophagy (ATG) depression are considered hallmarks of GBM. In fact, mTOR-dependent ATG inhibition strongly correlates with the presence of EVs, which in turn promotes glioblastoma cancer stem cells (GSCs) self-renewal, proliferation, and infiltration. ATG and exosome release are reciprocally regulated. In detail, a failure in ATG enhances exosomal release. Therefore, strategies aimed at targeting on mTOR-dependent extracellular vesicles could be a promising approach for GBM prevention and treatment.

**Abstract:**

Recently, exosomal release has been related to the acquisition of a malignant phenotype in glioblastoma cancer stem cells (GSCs). Remarkably, intriguing reports demonstrate that GSC-derived extracellular vesicles (EVs) contribute to glioblastoma multiforme (GBM) tumorigenesis via multiple pathways by regulating tumor growth, infiltration, and immune invasion. In fact, GSCs release tumor-promoting macrovesicles that can disseminate as paracrine factors to induce phenotypic alterations in glioma-associated parenchymal cells. In this way, GBM can actively recruit different stromal cells, which, in turn, may participate in tumor microenvironment (TME) remodeling and, thus, alter tumor progression. Vice versa, parenchymal cells can transfer their protein and genetic contents to GSCs by EVs; thus, promoting GSCs tumorigenicity. Moreover, GBM was shown to hijack EV-mediated cell-to-cell communication for self-maintenance. The present review examines the role of the mammalian Target of Rapamycin (mTOR) pathway in altering EVs/exosome-based cell-to-cell communication, thus modulating GBM infiltration and volume growth. In fact, exosomes have been implicated in GSC niche maintenance trough the modulation of GSCs stem cell-like properties, thus, affecting GBM infiltration and relapse. The present manuscript will focus on how EVs, and mostly exosomes, may act on GSCs and neighbor non tumorigenic stromal cells to modify their expression and translational profile, while making the TME surrounding the GSC niche more favorable for GBM growth and infiltration. Novel insights into the mTOR-dependent mechanisms regulating EV-mediated intercellular communication within GBM TME hold promising directions for future therapeutic applications.

## 1. Introduction

Gliomas are the most frequent intracranial tumors in adults [1]. Within this heterogeneous group of neoplasms, glioblastoma multiforme (GBM) represents the highest and most severe prognostic grade, namely “grade IV” glioma, according to the World Health Organization (WHO) classification system [2,3]. With a median overall survival of 14 months after diagnosis, GBM remains the most aggressive and lethal among all primary brain tumors [4].

In particular, GBM is featured by a marked intra-tumoral cellular heterogeneity, high proliferative rate, and extensive invasiveness within the surrounding healthy brain parenchyma [5,6,7,8]. Recent findings demonstrate that GBM malignant behavior is associated with the presence of a small subpopulation of cells referred to as glioblastoma cancer stem cells or glioma stem cells (GSCs) [9,10,11]. Remarkably, these cells display biological properties of normal neural stem cells, such as increased growth rate, enhanced self-renewal, and pluripotency [12,13]. Thus, GSCs represent the amplification of neural stem cell (NSCs), which reside within perivascular niches of the adult human brain [14,15]. The uncontrolled proliferation within these restricted neurogenic areas results in the establishment of a reservoir of tumorigenic cells forming the tumor bulk [16,17,18,19]. As occurring in many solid tumors, even GBM features a hierarchical organization, mirroring a normal stem cell system. In particular, a small subset of pluripotent and self-renewing GSCs stands at the apex of this hierarchy. The asymmetrical division of GSCs replenishes the pool of cancer stem-like cells, while giving rise to a population of phenotypically heterogeneous tumor cells. The more differentiated progeny cells, with low or no-tumorigenic potential, are restricted at the bottom.

Although numerous studies have revealed that GSCs originate from NSCs, emerging results suggest that GSCs enrichment may occur from a de-differentiation of normal brain cells [20,21]. For instance, recent experiments showed that epigenetic modifications can revert non-GSCs into GSCs [22]. Therefore, the issue of GBM cell(s) of origin is still on debate, providing a major complexity in understanding GBM neurobiology. At the same time, this hurdles for identifying a therapeutic strategy aimed at eradicating GSCs, which in turn contributes to the dismal prognosis of GBM patients.

High rate of tumor recurrence is a prominent feature of high-grade gliomas, and especially GBM. Unfortunately, GBM frequently recurs nearby surgical resection margin with lower response rate to conventional treatments [23]. Multiple studies have demonstrated that GSCs harbor high tumor initiating and clonogenic potential; thus, emerging as the driving force of GBM therapeutic resistance and relapse [24,25,26,27]. In particular, the remaining therapeutic-resistant GSCs can provide a reservoir of cells from which recurrent GBM arises. In fact, after debulking, these cells can migrate within the resection cavity, and initiate and recapitulate the whole tumor [28]. In addition, remaining GSCs show enhanced resistance to current treatments [29]. To date, management protocols for recurrent GBM (rGBM) patients are not well defined and most of rGBM are not eligible for surgical re-resection. 

**Figure 1:**
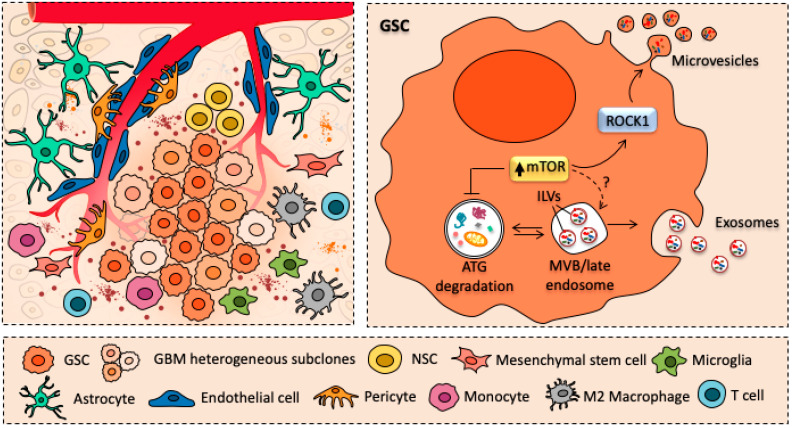
Glioblastoma-derived extracellular vesicles as mediators of intercellular communication. Cartoon schematizing the crosstalk between glioblastoma cancer stem cells (GSCs) and major cellular components of glioblastoma (GBM) tumor microenvironment (TME) surrounding GBM stem cell niche. In detail, GSCs can establish a dynamic and continuous interaction with resident (endothelial cells, pericytes, astrocytes) and infiltrating cells (macrophages, T cells) of TME by releasing extracellular vesicles (EVs), such as microvesicles and exosomes. Remarkably, the abundant release of EVs is tightly intermingled with mammalian Target of Rapamycin (mTOR) hyper-activation and subsequent autophagy suppression, which are key in promoting GSC self-renewal and proliferation.

Another level of complexity in the understanding of GBM biology is the ability of GSCs to modify the microenvironment surrounding the GSCs niche, making it more favorable to GBM growth and infiltration [30]. In fact, it has been demonstrated that GBM cells can interact with neighboring or distant cells, either tumorigenic and non-tumorigenic parenchymal ones, such as astrocytes, endothelial cells, pericytes and immune cells [31] (Figure 1). This may occur either through direct cell-to-cell contact (e.g., trogocytosis, tunneling nanotubes, gap junctions) or it may involve the release of extracellular vesicles (EVs), such as microvesicles and endosome-derived exosomes [31]. These latter recently emerged as key mediators of the crosstalk between GBM and tumor microenvironment (TME) [32,33]. 

In particular, GBM-derived EVs harbor diffusible pro-oncogenic cargoes, which once released within the extracellular milieu, can act both as autocrine and paracrine factors. For instance, GBM-released exosomes may contribute to GSCs niche’s maintenance by modulating GSCs’ stem cell-like properties while restraining them from differentiation [34,35,36]. Additionally, several studies demonstrate that EV-loaded astrocytes can elicit positive effects on GBM growth and proliferation [37,38,39]. Again, GBM-derived microvesicles modulate GBM-associated endothelial cells, thus, promoting tumor angiogenesis [34,40,41,42]. Emerging data also indicate that GBM release EVs to promote tumor immune escape [43]. For instance, GSC-derived exosomes can act on monocyte inducing a shift towards an immune suppressive phenotype, which in turn fosters tumor invasion and progression [44,45]. Moreover, internalization of GBM-derived EVs by CD14+ monocytes is involved in peripheral T cell immunosuppression [46].

Collectively these observations raise the possibility that EVs, and mostly exosomes, may act as biological vehicles, which can promote phenotypic and molecular reprogramming in neighboring recipient cells, either normal or tumoral, thus, posing cell-to-cell communication as an unconventional mechanism of disease spreading in GBM. 

Although the complexity of tumor-stroma interactions is not fully understood, strategies aimed at disrupting EV-based TME communication emerge as another potential therapeutic approach for antagonizing GBM. 

In keeping with this, recent publications revealed a potential role of the mammalian Target of Rapamycin (mTOR) in modulating the release of protein-enriched exosomes. Remarkably, this molecular complex, which controls cell growth and proliferation, is key in GBM biology since it is strongly relates with GSC loss of differentiation [26]. In fact, mTOR hyperactivation, which occurs concomitantly with autophagy (ATG) depression, is key in promoting GSC self-renewal, proliferation, infiltration as well as resistance to standard treatments [18]. In addition, dysfunctional ATG greatly increases neuronal release of exosomes and exosome-associated prions [47]. In contrast, pharmacological inhibition of mTOR promotes a phenotypic shift of GSCs towards a neuron-like phenotype, while interfering with exosome release. Given its potential ability to modulate EV-mediated intercellular communication, mTOR inhibitors may have tremendous potential as therapeutic agents for treating GBM [48].

Therefore, the first part of present review briefly summarizes the current state of knowledge regarding the role of EVs in cell-to-cell communication in the central nervous system (CNS) both in physiological and pathological conditions. Here, we discuss the role of the mTOR pathway in altering exosome-based cell-to-cell communication, which, in turn, modulate GBM infiltration and volume growth. In the second part, we discuss recent data about the role of mTOR-dependent, GBM-derived EVs as main drivers for GBM tumorigenicity, focusing on how EVs can modulate TME, as well as GSC stem cell niche.

## 2. EV-Mediated Cell-to-Cell Communication in CNS: From Physiological to Pathological Conditions

Intercellular communication is a physiological mechanism underlying cellular and systemic homeostasis. It involves intercellular transfer of signals, which propagate within microenvironment, between releasing and neighbor receiving cells. Remarkably, cell-to-cell communication occurs either through direct cell-to-cell contact or it can involve membrane-bound proteins [49,50,51,52]. In fact, signaling molecules: (i) are exposed on fragments of plasma membrane and exchanged between two cells (trogocytosis, [50]); (ii) are transported along intercellular tunneling nanotubes [51,52]; (iii) or they interact with trans-membrane receptor proteins. In recent years, a novel mechanism of intercellular communication has emerged [53]. This latter method of cell-to-cell communication enables a regular exchange between cells in a contact-independent manner via the release of membranous extracellular vesicles (EVs). These, in turn, are loaded with proteins, nucleic acids, and lipids as mediators of signal transduction. Although organelle terminology in literature is still confounding, the EVs are classified in three main groups, based on their biogenesis and size: apoptotic bodies (ABs), shedding microvesicles (MVs, or ectosomes), and exosomes [54]. ABs vary in size from 800–5000 nm in diameter, and they are released as blebs from dying cells, which undergo programmed cell death. MVs are membranous vesicles (50–1000 nm in diameter), which originate from budding of the plasma membrane of healthy cells and shed outward into the extracellular environment [55,56,57]. In contrast, exosomes are the smallest EVs (30–100 nm in diameter) and they originate from the endosomal system upon fusion of multivesicular bodies (MVBs) with the cell surface [58,59]. These endosome-derived vesicles are released from many cell types and they have been isolated from different body fluids, encompassing blood, saliva, and cerebrospinal fluid [60]. Remarkably, the release of exosomes is recruited in several physiological processes, such has angiogenesis, immune response, and antigen presentation [57]. Moreover, exosomes are key in brain development, synaptic transmission, myelin sheath formation, and nerve regeneration [61]. In fact, neurons, astrocytes, oligodendrocytes, and microglia release exosomes into the extracellular milieu; thus, affecting intercellular communication within the CNS [62].

Although the release of EVs is recruited in several physiological processes, its role in neurodegenerative disorders and CNS cancer have attracted particular interest. In fact, beside their physiological role in the clearance of unnecessary molecules, there is evidence that these secreted vesicles facilitate intercellular spreading of neurotoxic, prion-like proteins typical of neurodegenerative disorders, such as Parkinson’s and Alzheimer’s disease; thus, contributing to disease pathogenesis [63].

Moreover, recent findings have demonstrated that these vesicles act both as autocrine and paracrine factors, thus, participate in cancer pathogenesis, especially in CNS tumors, such as neuroblastoma, medulloblastoma, and specifically GBM [33,45,64,65]. In fact, EVs may serve as vehicles to transfer proteins and genetic material from tumoral towards healthy recipient cells to modulate their phenotype [38,39,41,44,45]. This is crucial in tumor biology, since tumor cells create a favorable microenvironment supporting their growth and survival. In the next paragraphs, we will discuss recent evidence on the multifaceted tumor-supporting role of EVs specifically in GBM.

## 3. The Role of mTOR in Exosome-Based Cell-to-Cell Communication

Exosomes are nano-sized (30–100 nm) membrane vesicles of endosomal origin that are generated by inward budding of the limiting membrane of multivesicular bodies (MVBs/late endosomes); thus, resulting in the formation of intraluminal vesicles (ILVs). Briefly, ILVs budding and MVB formation along with sorting of ubiquitinated proteins within ILVs are coordinated by the endosomal sorting complexes required for transport (ESCRT) machinery, which is composed of four multiprotein complexes (ESCRT-0 to III), and the VPS4 ATPase [66]. Upon fusion of MVBs with the plasma membrane, the enclosed ILVs (from now on referred to as exosomes) are released into the extracellular space. Alternatively, MVB cargoes can be degraded upon fusion with lysosomes. Being released from many types of eukaryotic cells, exosomes were initially proposed as a mean to secrete waste/unwanted material resulting from cell metabolism as well as potentially toxic molecules that may be harmful for the cell itself. However, over the last years, exosomes emerged in intercellular communication in normal physiological conditions as well as in pathological progression [67].

Recent studies revealed that exosome formation and release are intimately connected with the main cell clearing system of eukaryotic cells, namely autophagy (ATG). This evolutionarily conserved lysosome-mediated catabolic pathway ensures cell proteostasis by clearing unfolded/misfolded proteins; thus, preventing their accumulation, aggregation, and spreading [68,69,70]. The ATG pathway is initiated with the formation of a double-layered membrane vacuole named phagophore, which at early stages is not yet complete. Phagophore maturation and sealing leads to the formation of a specialized vacuole termed autophagosome, which stains for ATG gold standard markers namely beclin-1 and LC3 [71]. The autophagosomes encloses a variety of substrates (e.g., ubiquitinated proteins and whole organelles) that are subsequently shuttled to the lysosomal compartment. Fusion of autophagosomes with lysosomes generates a catalytic organelle, the autophagolysosome, leading to autophagic degradation and recycling of sequestered cytosolic cargoes [72,73,74,75]. When operating in baseline conditions, ATG ensures the maintenance of intracellular homeostasis by keeping steady several activities such as organelles and proteins turnover. Moreover, ATG can be either up- or down-regulated to meet specific cell needs. Thus, when a failure in ATG pathway occurs, the accumulation of misfolded proteins/damaged organelles overcome the attempt of ATG to digest them. This, in turn, further exacerbates ATG engulfment while promoting spreading of partially digested/undigested materials within healthy brain areas, as commonly described in several neurodegenerative disorders [76]. Remarkably, emerging evidence indicate that there is a substantial and coordinated interplay between ATG lysosomal degradation and exosomal release in response to the accumulation of cytosolic protein aggregates [77,78]. In fact, several reports indicate that ATG modulators can regulate MVBs formation and exosome secretion [79,80]. For instance, pharmacological inhibition of ATG with either 3-methyladenine (3MA) or bafilomycin A1 (BAF) leads to increased exosomal secretion [81,82,83]. Conversely, ATG induction with rapamycin promotes the fusion of MVBs with autophagosomes; thus, preventing extracellular release of exosome [47,84]. Among various protein kinases, which regulate ATG pathway, the best-known ATG inhibitor is the mechanistic Target of Rapamycin (mTOR). Remarkably, this serine/threonine kinase, which controls cell growth and proliferation, has critical roles in various pathological conditions encompassing neurodegeneration and brain tumors [18,85]. As witnessed by multiple experimental and pathological findings, mTOR upregulation and subsequent defective autophagy strongly correlate with GBM proliferation, relapse, and lethality. Constitutive activation of mTOR signaling and autophagy failure emerge as critical standpoint of GSCs self-renewal and proliferation. Moreover, GSCs cell migration and infiltration rely on the very same mTOR activation and ATG suppression [19,26]. Notably, beneficial effects mTOR inhibitors/ATG inducers have been demonstrated both in vitro and in vivo models of GBM [26,48,86,87,88,89,90]. For instance, rapamycin and its analogs (i.e., rapalogs) have shown to contribute in reducing GSC stem-like properties, while promoting differentiation, thus restraining GBM growth and infiltration. Thus, since ATG and exosome release are reciprocally regulated [83,91,92], mTOR-dependent ATG depression may enhance GBM exosome-dependent release of cytosolic cargoes. This may provide an explanation for the observation that GSCs are characterized by an abundant release of exosomes enriched in proteins, mRNAs, and miRNAs [34] (Figure 1). Hence, GSCs can benefit from mTOR-dependent ATG suppression since it may enable exosome-mediated disease spreading. On the other hand, preventing exosomal release by inducing ATG with mTOR inhibitors may provide a therapeutic strategy for GBM treatment.

Although the molecular mechanisms underlying the role of mTOR in modulating the release of protein-enriched exosomes still remains largely unexplored. A very recent paper reported that activation of mTOR signaling induced the release of MVB-derived exosomes and microvesicles (MVs) through the activation of Rho-associated protein kinase (ROCK) signaling [93] (Figure 1). Remarkably, Rho GTPase and the associated protein ROCK are important regulators of actin dynamics and are required for membrane budding and vesicle biogenesis. In fact, RhoA promotes exosome biogenesis and MVs release from cancer cells, through the activation of ROCK kinase [94,95]. Although evidence reported so far reveal a complex link between mTOR, ATG, and exosome in GBM neurobiology, such an issue requires further investigations being critical to understand exosome-based GBM growth and propagation throughout the whole CNS.

## 4. The Role of mTOR-Dependent GSCs-Derived EVs

A predominant feature of GBM is the ability to recruit and exploit neighbor non-tumorigenic cells to support tumor growth and progression. GBM interacts with several cell types, encompassing endothelial cells, perivascular pericytes, astrocytes, and immune cells [31]. This intercellular communication may occur in various ways, either through a direct cell contact (i.e., tunneling nanotubes, gap junctions) or through cell-to-cell transfer of insoluble cargoes via the release of EVs, such as microvesicles (MVs) and exosomes [34,40,96,97]. Although EVs may differ in morphology and biological effects, they represent a unique way of long-distance, contact-independent, mechanism of intercellular communication, acting as mediators in the crosstalk between GBM and the surrounding TME.

In 2008, a pioneer study by Skog and colleagues revealed that GBM cell lines produce microvesicles varying in size from about 50–500 nm [34]. By means of scanning EM, the authors documented the abundant shedding of MVs on the surface of cultured human GBM primary cells. GBM cells in culture release more than twice MVs per cell over 48 hours when compared with normal human fibroblast [98]. Again, circulating MV levels are significantly increased in cerebrospinal fluid (CSF) and serum of GBM patients compared with healthy volunteers [99,100].

A further step in the investigation was to determine the content and biological function of GBM-derived EVs. In particular, these authors demonstrate that (i) tumor cell-derived MVs contain a variety of mRNAs and miRNAs; (ii) mRNA-loaded MVs can be delivered from GBM cells into other recipient cells, such as human brain microvascular endothelial cells (HBMVECs); (iii) mRNAs carried by tumor cell-derived MVs can enter HBMVECs and generate a functional protein; thus, providing evidence that MVs cargoes can easily modify the translational profile of recipient cells. Since the relevant study by Skog and colleagues [34], increasing evidence shows that GBM cells can transfer a plethora of information. This encompasses proteins, RNA (i.e., mRNAs, miRNA, ncRNAs), retrotransposons, and other DNA elements [34,41,100,101,102,103].

**Figure 2:**
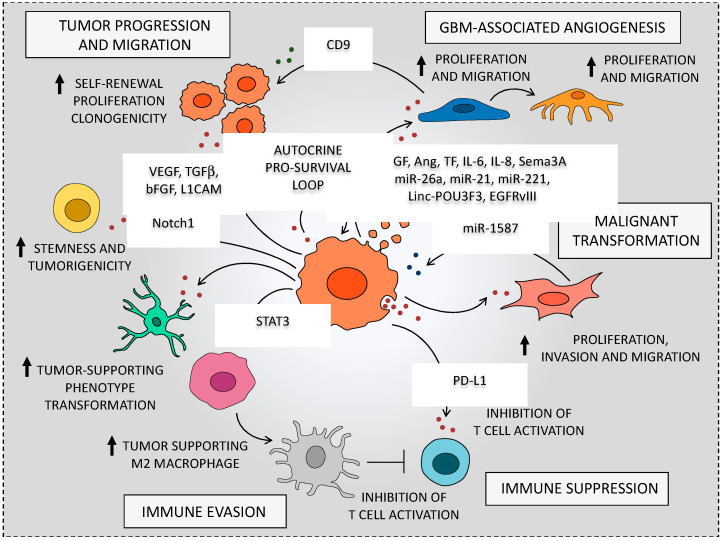
Glioblastoma-derived extracellular vesicles can actively remodel the tumor microenvironment. The cartoon summarizes the major effects of glioblastoma-derived extracellular vesicles (EVs) on cells from the tumor microenvironment. Messages carried in exosomes, such as proteins, RNA and miRNAs, can be taken up by TME recipient cells to elicit a phenotype modulation towards a tumor-supporting phenotype. For instance, GBM-derived exosomes have shown to modulate endothelial cells and pericytes, thus promoting tumor angiogenesis. Again, GSC-derived EVs recruit immune cells (monocytes, macrophages, T cells) to drive immune modulation that fosters tumor growth. Moreover, EVs can be released within the extracellular milieu just close to the perivascular niche thus acting as autocrine factors to promote GSC self-renewal and proliferation. Therefore, EVs can be employed both in autocrine and paracrine modes by GSCs to give a remarkable support to GBM survival and malignant progression.

Once taken up by neighbor recipient cells, these bioactive molecules can trigger several biological effects aimed at inducing tumor-supporting functions in neighbor non-tumorigenic cells. For instance, GSC-secreted EVs can act as paracrine stimuli to induce endothelial cell activation and thus actively promoting angiogenesis [34,40,41,42]. Emerging data also indicate that exosomes participate in GSC immune evasion, since they are able to modify the phenotype of monocytes, macrophages, and lymphocytes. This enables GBM to evade immune-surveillance, while further enhancing tumor survival, infiltration, and therapeutic resistance [43,44,45,46]. Again, EVs released within the extracellular milieu, just close to the perivascular niche, can promote GSC self-renewal and proliferation along with transformation of NSCs into tumor-promoting cells [34,35,36,37,38,39] (Figure 2).

In the next paragraphs, we provide evidence on how GSC-derived EVs, acting both in autocrine and paracrine ways, create an optimal microenvironment for GBM survival and malignant progression.

## 5. mTOR-Dependent GSC-Derived EVs Promote GBM Angiogenesis 

Compelling evidence demonstrate that GBM-derived EVs provide intense growth signals that markedly enhance brain endothelial cell (EC) proliferation to foster tumor vascular network formation (Figure 2). For instance, Liu et al. [99] provided evidence that GBM-derived MVs significantly induce angiogenesis by enhancing Akt/beta catenin-dependent cell proliferation and migration of ECs [99]. This issue is of considerable interest in glioma malignancy since intense and aberrant angiogenesis is crucial to provide nourishment to GSCs [14,15]. In both in vitro and in vivo studies, it was found that GSCs release substantial amount of EVs to elicit angiogenesis. In particular, the interaction of EVs-expressed membrane proteins with EC surface receptors promotes specific intracellular angiogenetic signaling cascade. For instance, Kucharzewska et al. [42] showed that GBM-derived exosomes activate ECs surface receptors, such as epidermal growth factor receptor (EGFR), ephrin type A receptor 2 (EPHA2), and vascular endothelial growth factor receptor 2 (VEGFR2), which in turn stimulate major intracellular kinase pathways (i.e., ERK1/2 MPK, PI3K/ATK, FAK). EVs-driven modulation of ECs may also be produced independently of receptor-dependent intracellular cascades. In fact, this may occur through the internalization of GSC-secreted vesicles within the recipient cells [104].

The pro-angiogenic effect of GBM MVs was associated with several angiogenic proteins, such as vascular endothelial growth factor (VEGF), angiogenin, interleukin-6 (IL-6), IL-8, tissue factor (TF), tissue inhibitor of metalloproteinase-1 (TIMP-1) and TIMP-2 [33,34,40,42,105,106,107,108]. For instance, GSC-EVs loaded with VEGF initiate angiogenesis in brain endothelial cells, thus, supporting GSCs perivascular niche. In fact, EVs-mediated endothelial cell sprouting was impaired in VEGF-A-depleted GSCs [33].

Likewise, EVs-harbored TF could act as paracrine signals to sustain ECs proliferation and migration, while antibody-mediated TF blockage occludes their activation [40]. In fact, elevated TF released within microvesicles with exosome-like features, triggers protease-activated receptor 2 (PAR-2) up-regulation in hypoxic ECs. Induction of PAR-2/ERK1/2 signaling increases levels of the pro-angiogenic growth factor heparin-binding EGF-like growth factor (HB-EGF) [40]. Again, cell migration and tube formation ability of human brain microvascular endothelial cells (HBMVECs) is increased upon EVs stimulation. As demonstrated by an in vitro angiogenesis assay, HBMVECs incubated in medium supplemented with GBM MVs had two-fold increase in tubule length compared with cells grown in endothelial basal medium [34].

Along with pro-angiogenic potential, VEGF-A derived from GSCs-secreted vesicles also increases endothelial permeability, which is another pathological hallmark of GBM. In particular, hypoxic GBM-derived exosomes carrying VEGF-A enhance blood–brain barrier (BBB) permeability by specifically altering the expression of claudin-5 and occludin in ECs, in both in vitro and in vivo GBM models [108]. In fact, pro-permeability effect was completely abolished in patient-derived GSCs transfected with anti-VEGF-A siRNA [33].

In line with this, reducing Semaphorin3A (Sema3A) availability in GSCs-released EVs significantly hamper brain ECs permeability [106,109], thereby disrupting brain vascular integrity. Altogether, these observations feed the hypothesis that GBM actively remodels brain vessels to promote survival and infiltration via an EV-mediated mechanism. In addition, GBM can take advantage of altering BBB integrity to limit site-specific drug delivery to tumor, which may ultimately have profound consequences on the survival of GBM patients.

In addition to pro-angiogenic proteins, GBM tumor cells can deliver a plethora of genetic information to neighbor ECs. In fact, EVs released from GSCs contain mRNAs, microRNAs (miRNAs), non-coding RNAs (ncRNAs), as well as genomic DNA and cDNAs. All these nuclei acid can be transferred to ECs [34,98,103,110,111,112,113,114]. Remarkably, miRNAs derived from hypoxic CD133+ U87 glioblastoma cells emerged as powerful inducers of tumor vasculature and glioma cells proliferation in vitro [115]. Once taken up by recipient cells, these nucleic acids can trigger several effects in ECs, including epigenetic modifications [41,42,114,116].

For instance, GSCs-derived exosomes are highly enriched in miR-21, which can promote the angiogenic ability of ECs by stimulating the VEGF/VEGFR2 pathway [117]. Likewise, EVs-delivered miR26a promotes angiogenesis of HBMECs by up-regulating VEGF, through the activation of the PI3K/Akt pathway, via PTEN inhibition [118]. Similarly, exosomal miR-221 from GSCs markedly enhances endothelial tube formation and proliferation [107]. Again, exosome-mediated transfer of long non-coding RNA POU3F3 (linc-POU3F3) from GSCs to brain ECs can promote angiogenesis. In fact, once rapidly internalized by HBMECs, these exosomes enriched in linc-POU3F3 stimulate angiogenesis-related gene and protein expression (i.e., VEGFA, bFGF, bFGFR, and Angio), thus, promoting in vitro HBMECs migration, proliferation, tubular-like structure formation and in vivo angiogenesis [113].

GSCs-derived EVs contribute to intercellular transfer of the oncogenic receptor EGFRvIII towards brain ECs [101]. This is of particular interest since EGFR gene mutations/amplifications are frequent in GBM samples [119]. In particular, EGFRvIII-containing exosomes derived from GBM cells induce several morphological and phenotypic changes in endothelial recipient cells, encompassing the activation of MAPK and Akt signaling, the increase in anchorage-independent growth capacity and changes in VEGF, Bcl-x(L), and p27 gene expression [101].

Although evidence reported so far demonstrated a unidirectional EV-mediated transfer from GSCs towards ECs, recent intriguing studies implicated glioma-associated parenchymal cells to promote GBM tumorigenicity via exosome. In fact, EVs carrying morphogens, growth factor, and nucleic acids are transferred from ECs towards GSCs to establish a bidirectional interaction between GBM tumor cells and vascular system. It was recently emerged that EC-derived EVs transfer of CD9 increases GSC self-renewal and proliferation in vitro, as well as GSC tumorigenicity in vivo [120]. Remarkably, CD9 is a transmembrane protein known as a biomarker for GSCs and it is involved in tumor cell proliferation, survival, and invasion [121,122].

Taken together, these findings indicate that EVs represent major contributors to GBM angiogenesis. In fact, EV-delivered soluble cargoes and genetic information may work as paracrine factors to induce dynamic and complex tumor vascular re-arrangements. Remarkably, GBM EV-mediated intercellular communication emerged as a pivot to promote GSCs/ECs interactions, while making GSC microenvironment more favorable to GBM growth and infiltration.

Therefore, extensive research is required to decipher further each contributor within the myriad of pro-angiogenic molecules delivered within glioma EVs. Moreover, a more in-depth investigation elucidating the molecular mechanisms of these GSC EV-delivered cargoes may disclose potential therapeutic targets to combat GBM.

## 6. mTOR-Dependent EVs Effects on Glioma-Associated Parenchymal Cells 

An extensive body of work provided evidence that GSCs receive proliferation signals overcoming growth-inhibition, which is concomitantly operated from different cell components within the brain TME, including ECs, glia, or neurons [19]. Besides different GBM tumor cell types (i.e. GSCs, progenitor cells, tumor non-stem cells), GSC perivascular niche harbor several non-tumorigenic, stromal cells. In fact, intermingled with GBM tumor mass, astrocytes, ECs, pericytes, resident, and infiltrating immune cells represent major non-cancerous cellular constituents of GBM TME. Remarkably, these cells are the main targets for EVs released from GSCs (Figure 2). Furthermore, EVs released by surrounding stromal cells maintain a supportive niche, which enhances GSC proliferation and aggressiveness [97]. Therefore, these cells are not mere bystanders, but they emerge as active players in GBM TME adaptive remodeling by establishing a dynamic EV-mediated crosstalk with GSCs [123].

### 6.1. The Role of Astrocytes

An EV-driven tumor-supportive function is described in peritumoral normal astrocytes. Increasing evidence indicate that astrocytes can shed EVs containing several neuroprotective molecules and neurotrophic factors, which can exert a proliferation-promoting effect on glioma cells. For instance, secretion of CD44 ligand osteopontin by stromal astrocytes can enhance a stem cell-like phenotype in glioma cells, while promoting GBM growth in vivo [124].

Moreover, paracrine factors secreted from astrocytes were shown to induce the expression of several migration-associated genes in GSCs [125]. Vice versa, exposure to GSC EVs elicits a phenotypic modulation of recipient astrocytes, towards a pro-inflammatory, tumor-supporting phenotype. In particular, GBM EV-treated astrocytes release great amount of growth factors (i.e. VEGF, EGF, FGF) and chemokines (i.e., CXCL1, 9,10) within the extracellular milieu, which generates a growth-stimulating medium for GBM tumor cells [38]. At the same time, GBM-EV stimulates astrocytes, which feature an increased number of podosome. These cells operate extracellular matrix degradation, which support GBM invasion and infiltration [39]. In addition, as evidence by the secretome assay, GBM EVs uptake by astrocytes increases anti-inflammatory cytokines production, thus suppressing local immunity [38].

Notably, GSC EVs induces profound modifications in major signaling pathways, such as TP53 signaling inhibition and MYC activation, which could drive recipient astrocytes towards a tumorigenic phenotype [38,39]. Remarkably, these phenotype changes lead to astrocytic end-feet displacement leading to a disruption of astrocyte-vascular coupling and thus, BBB breakdown [126].

Altogether, these data indicate that EVs participate in GSC/astrocytes reciprocal modulation of expression and translational profile, thus posing EV-mediated cell-to-cell communication as an unconventional mechanism of glioma transformation and disease spreading.

### 6.2. The role of Perivascular Pericytes

In keeping with the role of EVs in GBM neurobiology, a tumor-supportive function has been described in perivascular pericytes, which represent an important component of GBM vasculature. In fact, due to their close relationship with ECs, they contribute to vessel integrity and function [127]. In addition, they facilitate GBM evasion of immune surveillance [128,129,130]. In particular, GBM-derived hypoxic EVs exerts a paracrine stimulation of pericyte proliferation (via PI3K/AKT pathway) and migration, which contribute to tumor vasculature stabilization [42]. Remarkably, this effect was potentiated by pre-conditioning ECs with GBM derived exosomes. In fact, ECs stimulated with GBM-derived exosome enhances pericyte migration and PI3K/AKT signaling activation by specifically secreting growth factors and cytokines within TME [42].

Although the mechanisms implicated in EV-mediated modulation of BBB integrity are yet unexplored, this issue is of particular interest in GBM neurobiology since it may be a potential area of therapeutic intervention. Unraveling the role of EVs in the dynamic crosstalk between GBM cells, ECs and pericyte could help to decipher how GBM can actively reshape tumor vasculature, which ultimately serves to promote GBM growth, invasion and therapy refractoriness. Therefore, strategies aimed at interfering with EV-mediated crosstalk between GBM and vascular cells will hopefully contribute to ameliorate GBM patients’ outcome.

### 6.3. The Role of Mesenchymal Stem Cells (MSCs) 

Apart from ECs and pericytes, recent experimental findings demonstrate that other non-tumoral cells, which reside in the perivascular niche, are pivotal in GSCs maintenance and survival by mechanisms depending on EVs secretion [131]. This is the case of stromal cells resembling mesenchymal stem cells (MSCs), which recently emerged as a novel stromal component of GBM TME. MSCs, which were originally isolated from bone marrow, represent adult multipotent progenitor cells, with a remarkable self-renewal potential, which differentiate into mesodermal (i.e. adipocytes, chondrocytes, and osteoblasts) and non-mesodermal cell lineages [132]. Recent intriguing reports implicate MSC-derived exosomes in GBM maintenance. In fact, MSCs represent a newly identified source of growth factors and morphogens that increase GSCs self-renewal, while contributing to tumor growth and tropism [133]. For instance, MSCs release interleukin-6 (IL-6) which can induce pro-tumorigenic effects on GBM by increasing GSC stemness, proliferation and clonogenicity [131].

A recent report demonstrated that exosomes derived from glioma-associated (GA) mesenchymal stem cells (MSCs; (GA-MSCs)) enhance GBM aggressiveness in vitro by specifically increasing GSC proliferation and clonogenicity [112]. This effect was associated, at least in part, with exosome-mediated transfer of miR-1587, which downregulates the tumor-suppressive nuclear receptor co-repressor NCOR1 in the recipient GSCs [112]. These effects were confirmed in vivo in orthotopic xenografts. In fact, mice implanted with GSCs pre-treated with exosomes from GA-MSCs developed a greater tumor burden and possessed decreased survival compared with mice injected with untreated GSCs [112]. Again, another study published by Del Fattore et al. (2015) [134] provided evidence of the pro-tumorigenic activity of adipose MSC-derived exosomes in glioblastoma. However, the role of MSC in GBM tumorigenesis is still controversial, since there are conflicting reports on whether MSC-derived exosomes promote or suppress GBM growth, proliferation, and migration [135,136,137].

Notably, increasing evidence demonstrate that GSCs-derived EVs contribute to glioma-induced recruitment and migration of MSCs towards TME to take part in GBM propagation and dissemination [138,139]. In fact, exosomes derived from GSCs induce a tumor-like phenotype in human bone marrow MSCs (hBMSCs) by promoting cell proliferation, invasion, and migration [140]. In particular, proteomics and Western blot analyses demonstrate that GSCs-derived exosomes upregulate the protein levels of the proto-oncogene myc (C-myc), while downregulating the mRNA and protein levels of two main cell cycle regulators, namely P16 and P21, thus significantly enhancing cell growth and proliferation of hBMSCs. Moreover, migration and invasion increase in exosome-treated hBMSCs, as showed by the up-regulation of genes encoding matrix metalloproteinases (MMPs) MMP-2 and MMP-9 [140]. Collectively these data further confirm that GSCs profit from exosomes to modify gene expression within neighboring cell. In this way, GBM actively remodels its microenvironment to self-benefit. 

## 7. The Emerging Role of mTOR-Dependent EVs in Immune Escape 

Recently, there has been growing interest in whether GBM may hijack EV-mediated intercellular communication to evade immune-surveillance and promote GBM growth and infiltration [43]. Emerging data indicate that GSC-derived exosomes may exert an immunosuppressive effect by altering the phenotype of monocytes, macrophages, and microglia [46,141]. Although these latter represent different cell types, they are all referred as tumor-associated macrophages (TAMs), since they represent the major, non-tumorigenic, innate immune cell population residing within GBM TME. Recent in vitro studies revealed that GSC-EVs induce a phenotypic switch within monocyte precursors towards the immune-suppressive, tumor-supporting M2 macrophage phenotype [44,45]. Once internalized within monocytes, these GSC-derived exosomes produce cytoskeleton-morphological changes, increased phagocytosis, along with an increase in cytokine secretion, namely the chemokine (C-X-C motif) ligand 1 (CXCL1) and the monocyte-chemotactic protein 3 (MCP-3). Again, the transfer of the signal transducer and activator of transcription 3 (STAT3) from GSC-exosomes towards monocytes up-regulates programmed death ligand 1 (PD-L1). This latter exosome-mediated effect is of particular interest, since it suppresses T cell-associated immune response trough the interaction with the programmed cell death 1 (PD-1) receptor, which is expressed on the surface of activated T cells [45]. In addition, GBM can also evade T cell-mediated killing by shedding EVs decorated with PD-L1, thus, blocking T cell activation [142].

## 8. The Autocrine Role of mTOR-Dependent EVs on GBM Stem Cell Niche

Although EVs-based signaling is often regarded as a way for generating long-distance cell-to cell communication, it may play a role also in the “milieu” nearby GSCs. Beside the paracrine effects on endothelial and parenchymal/stromal cells. GSC-derived EVs contributes to the maintenance of cancer stem cell niche by acting as autocrine factors on GSCs themselves [143] (Figure 2). For instance, EVs-dependent release of VEGF promotes GSCs self-renewal and tumorigenesis [144]. This autocrine stimulation of GSCs relies on VEGFR2 receptor tyrosine kinase rather than VEGFR1 [145]. In fact, VEGFR2 is highly expressed on the surface of GSCs, where it mediates VEGF-dependent pro-survival signaling through the VEGF-VEGFR2-Neuropilin 1 (NRP1) pathway. In fact, either a direct inhibition of VEGFR2 tyrosine kinase activity and/or a shRNA-mediated knockdown of VEGFR2 or NRP1 dramatically decrease GSC cell viability [144]. Thus, GSC-EVs transfer of VEGF is pivotal to activate persistently intracellular pro-survival pathways that sustain GSC stemness properties.

Apart from VEGF, several factors derived from GSC-EVs have shown to promote GSCs self-renewal and proliferation, acting as autocrine factors [146]. These encompasses TGFβ, bFGF, Notch1, L1CAM, and axon guidance molecules, such as semaphorins (Sema) [146,147,148,149,150,151]. For instance, endogenous Sema3C is involved in GSC survival and stemness via Plexin-A2/D1-mediated Rac1/NF-kB signaling [150]. In fact, Sema3C is co-expressed with its receptors Plexin-A2/PlexinD1 in GSCs, whereas they are not expressed in NSCs, suggesting the presence of an autocrine pro-survival loop within the cancer stem cell compartment. GSC-derived exosomes support GBM infiltration by inducing a pro-migratory activation of GBM cells [42]. For instance, exosome-dependent release of Sema7A enhances GSC motility via activation of β1-integrin/FAK signaling [152]. Again, GBM-secreted Sema3A act as an autocrine migration-promoting stimulus on GSCs [153,154,155] through the binding to NRP1/PlxA1. In contrast, depletion of secreted-Sema3A by RNAi reduces GSCs diffusion, thus limiting GBM infiltration [153].

At the same time, GSC secreted vesicles diffuse throughout the TME to mediate phenotype reprogramming of NSCs and non-GSC glioma cells surrounding the perivascular niche. For instance, in vitro analysis indicates that GSC-derived EVs transforms NSCs into tumor-promoting cell, with enhanced proliferation, migration, and clone-formation [156]. Non-GSC glioma cells when loaded with Notch1 filled exosomes trans-differentiate into GSCs [151]. In fact, GSCs released exosomes act at multiple levels to promote Notch1 signaling activation, stemness, and tumorigenesis. Altogether, these findings lead to consider the chance that GSCs hijack EV-mediated intercellular communication for self-maintenance, thereby facilitating tumor initiation and growth.

## 9. Conclusions 

The evidence discussed here converges in that GSC-derived EVs can be regarded both as autocrine and paracrine tumor-promoting and phenotype-transforming stimuli, which drive GBM malignant phenotype. In particular, EVs emerge as a powerful ancestral biological tool acting to modulate TME in relations to fostering or counteracting GBM cells fate at the expense or in favor of normal brain parenchymal cells. However, this raises critical questions as to which GBM-derived EVs, should be targeted as key drivers of glioma stem cell niche maintenance, tumor infiltration, immune evasion and therapeutic resistance. In fact, GBM-derived EVs may sort opposite effects. Thus, disclosing the nature and mechanisms by which EVs may potentially regulate GSCs self-renewal, proliferation and differentiation is expected provide critical information in the neurobiology of GBM to contribute in developing effective therapeutic interventions. 

Since GSCs are held responsible for tumor initiation, infiltration, and therapeutic resistance, targeting EV-mediated interaction between GSCs themselves and other normal cells within TME may provide a novel strategy to improve GBM patient outcomes.

### Outstanding Questions

In the last decade, EVs emerge as key players for GBM initiation, enlargement, and infiltration. In fact, GBM-derived diffusible cargoes can be propagated through EVs to alter the phenotypes of neighboring recipient cells, thus creating a favorable microenvironment for tumor growth and proliferation. For instance, exosome-mediated transfer of pro-oncogenic factors elicits an angiogenic, tumor-supporting phenotype in brain endothelial cells. Again, tumor immunosuppressive functions have been recently attribute to GBM-derived EVs.

Given their ability to actively reshape GBM microenvironment, dissecting the functional role of EVs in specific steps of GBM development and progression will provide potential therapeutic targets for GBM treatment. Furthermore, studies focusing on the correlation between EVs content and disease progression, will uncover their potential as GBM biomarkers; thus, improving GBM early diagnosis and disease monitoring. Remarkably, since GBM was shown to benefit from mTOR-dependent ATG suppression and, thus, enhanced exosomal release, disclosing the molecular mechanisms by which mTOR regulates EV-mediated intercellular communication within GBM tumor microenvironment will expand our understanding of glioma transformation and disease spreading. At the same time, investigations on mTOR-mediated EVs release will provide novel approaches for drug development and future therapeutic interventions.

## Abbreviations

BBB	Blood–brain barrier
CNS	Central nervous system
CSF	Cerebrospinal fluid
CXCL1	Chemokine (C-X-C motif) ligand 1
ECs	Endothelial cells
EGFR	Epidermal growth factor receptor
EPHA2	Ephrin type A receptor 2
EVs	Extracellular vesicles
GA-MSCs	Glioma-associated (GA) mesenchymal stem cells (MSCs)
GBM	Glioblastoma multiforme
GDEs	GSC-derived exosomes
GSCs	Glioma stem-like cells
HB-EGF	Heparin-binding EGF-like growth factor
hBMSCs	human bone marrow MSCs
HBMVECs	human brain microvascular endothelial cells
IL-6	Interleukin-6
IL-8	Interleukin-8
MCP3	Monocyte-chemotactic protein 3
miRNAs	microRNAs
MMPs	Matrix metalloproteinases
MSCs	Mesenchymal stem cells
MVs	Microvesicles
MVBs	Multivesicular bodies
NSCs	Neural stem cells
NRP1	Neuropilin
ncRNAs	noncoding RNAs
PD-1	Programmed cell death-1
PD-L1	Programmed death-ligand 1
plxA1	PlexinA1
rGBM	Recurrent GBM
Sema	Semaphorins
Sema3A	Semaphorin3A
Sema3C	Semapahorin3C
STAT3	Signal transducer and activator of transcription 3
TAMs	Tumor-associated macrophages
TF	Tissue factor
TIMP-1	Tissue inhibitor of metalloproteinase-1
TIMP-2	Tissue inhibitor of metalloproteinase-2
TME	Tumor microenvironment
VEGF	Vascular endothelial growth factor
VEGFR2	Vascular endothelial growth factor receptor 2
